# Data Quality Assessment, Representativeness, and External Consistency of a Coastal Oceanographic Buoy Using Copernicus Marine and ERA5 Products

**DOI:** 10.3390/s26185873

**Published:** 2026-09-16

**Authors:** Francisco Portillo, Nadia Rotbi, Nicolas Padilla, Consolacion Gil, John Alexander Taborda, Alfredo Alcayde, Maria Isabel Saez

**Affiliations:** 1 Department of Engineering, University of Almeria, CIAIMBITAL, 04120 Almeria, Spain; nrp590@inlumine.ual.es (N.R.); aalcayde@ual.es (A.A.); 2 Department of Computer Science, University of Almeria, CIAIMBITAL, 04120 Almeria, Spain; npadilla@ual.es (N.P.); cgilm@ual.es (C.G.); 3 Department of Electronic Engineering, University of Magdalena, CEIMAR, Santa Marta 47004, Colombia; jtaborda@unimagdalena.edu.co; 4 Department of Biology and Geology, University of Almeria, CEIMAR, 04120 Almeria, Spain; mabelsaezcasado@ual.es

**Keywords:** Coastal oceanographic buoy, Copernicus Marine Service, ERA5 reanalysis, oceanographic sensors, sensor data quality, significant wave height, subsurface temperature

## Abstract

Coastal oceanographic buoys provide high-frequency observations, but their scientific use requires channel-specific quality assessment and explicit treatment of representativeness. This study evaluates a coastal buoy deployed off Cabo de Gata, southeastern Spain, during two non-contiguous observation windows in 2025 and 2026. Buoy records were standardized, screened with quality-control tests, aggregated daily without gap filling, and compared with Copernicus Marine and ERA5 products as an external-consistency assessment. Gridded products were interpolated to the mean buoy position using inverse-distance weighting, and Copernicus Marine temperature was vertically interpolated to the 5 m and 10 m buoy-sensor depths. Completeness was 88.1–88.3% in 2025 and 99.9–100.0% in 2026, with no gross-range failures. Significant wave height and atmospheric pressure showed strong external consistency, with root mean square errors of 0.15/0.14 m and 0.63/0.59 hPa, respectively. Temperature at 5 m showed moderate-to-strong agreement, with errors of 0.98/0.64 °C. Wave-spectrum-derived wind speed showed larger, sea-state-dependent differences, although disagreement decreased under higher wave steepness. The workflow distinguishes channels suitable for daily environmental interpretation from those requiring additional metadata or independent field verification.

## 1. Introduction

Autonomous oceanographic buoys have become integral components of coastal observing systems, providing high-frequency in situ observations of sea state, upper-ocean seawater temperature, wind, atmospheric pressure, and platform status in areas where ship-based surveys are episodic and gridded products may not resolve local spatial variability [1,2]. Advances in instrumentation, telemetry, and low-power data acquisition have extended their role from isolated monitoring devices to operational platforms for model evaluation, hazard detection, and coastal decision support [3,4]. However, the scientific and operational value of these records depends on explicit data-quality procedures, because marine measurements can be affected by sensor exposure and drift, platform motion, biofouling, power limitations, and communication interruptions [5].

For coastal buoy deployments, data quality cannot be established through physical plausibility checks alone. Measurements may remain within acceptable ranges yet still be unsuitable for quantitative interpretation or external comparison due to temporal gaps, changes in sampling frequency, uncertain sensor exposure, or discrepancies between the quantity measured by the buoy and that represented by an external product [6,7]. These limitations are particularly relevant when point observations are compared with gridded models, reanalysis, or satellite-derived products, which represent spatially and temporally aggregated fields. A robust assessment should therefore treat each measurement channel separately and distinguish internal quality control (QC) (completeness, physical plausibility, and consistency) from external-consistency assessment against independent observational or gridded products.

Operational marine and atmospheric products provide useful external context for assessing buoy data. The Copernicus Marine Service integrates observations and numerical models to provide regional ocean products. In contrast, the European Centre for Medium-Range Weather Forecasts (ECMWF) fifth-generation atmospheric reanalysis (ERA5) provides a physically consistent atmospheric reanalysis [8,9]. Mediterranean wave-forecasting systems have also been evaluated against observational datasets, supporting their use to compare significant wave height (Hs) and related sea-state variables [10]. However, these products represent gridded analysis, forecast, or reanalysis fields rather than co-located in situ measurements. Differences in spatial support, effective measurement depth or height, temporal aggregation, and variable definition can therefore produce discrepancies even when both sources are internally consistent.

Although international observing infrastructures and best-practice initiatives have established broad principles for sustained observations, interoperability, metadata management, and QC [11,12], applying these principles to local or project-driven coastal deployments remains challenging. Such deployments are often evaluated over relatively short periods and may lack redundant in situ instrumentation, complete deployment metadata, or nearby reference stations. The practical gap is therefore not the absence of individual QC procedures, but the need to integrate channel-specific completeness, physical plausibility, internal consistency, and external-product comparisons into a coherent diagnosis of the platform. This distinction is important because agreement for one variable cannot be extrapolated to the remaining channels, and observed discrepancies may reflect measurement configuration or spatial representativeness rather than sensor malfunction.

In this context, the present study evaluates two non-contiguous observation windows of the Calibration and Geospatial Analysis of Temporal Oceanographic Anomalies (C-GATA) coastal oceanographic buoy off Cabo de Gata, southeastern Spain. The analysis combines internal QC with external-consistency assessment against Copernicus Marine and ERA5 products for seawater temperature at 5 and 10 m depth, wave parameters, wave-spectrum-derived wind speed and direction estimates, and atmospheric pressure. The objectives are threefold: (i) to quantify channel-specific temporal completeness and identify anomalous or inconsistent records; (ii) to assess agreement between buoy observations and gridded products using variable-specific scalar and circular metrics; and (iii) to determine which measurement channels are suitable for environmental interpretation and which require additional metadata, calibration, or independent in situ verification. By applying the same procedure to two separate observation periods, the study provides a reproducible channel-specific framework for diagnosing the quality and comparability of multi-sensor coastal buoy data.

The remainder of the paper is organized as follows: Section 2 describes the buoy, external products, and quality-assessment workflow; Section 3 presents the channel-specific external-consistency and diagnostic results; Section 4 discusses variable-specific confidence and limitations; and Section 5 summarizes the main conclusions.

## 2. Materials and Methods

### 2.1. Study Area, Buoy Platform, and Measurement Channels

**Figure 1:**
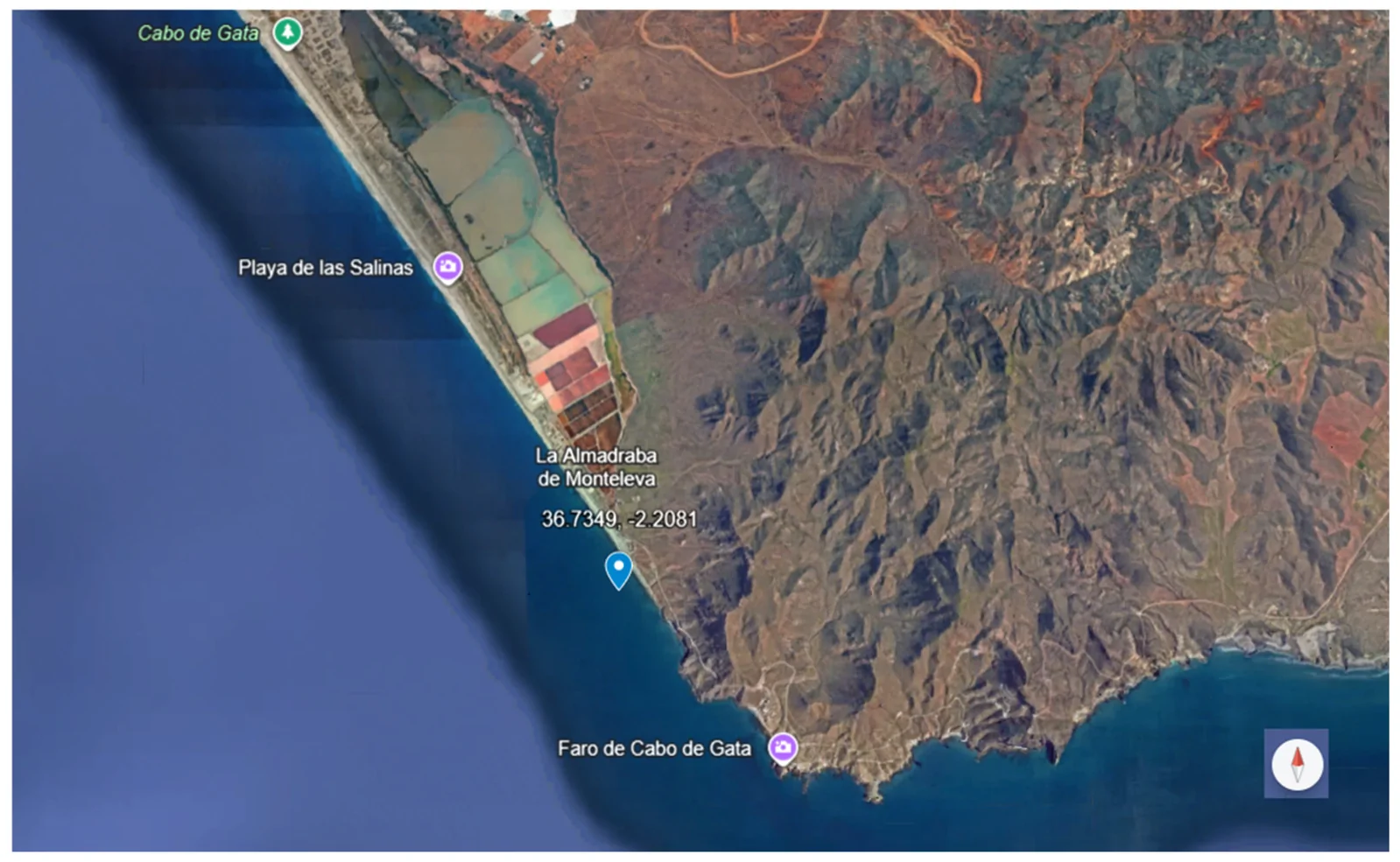
Location of the C-GATA buoy deployment site off Cabo de Gata, southeastern Spain. The marker indicates the mean buoy position during the analyzed observation windows (36.7349° N, 2.2081° W).

The C-GATA observing system was deployed off Cabo de Gata, on the southeastern Mediterranean coast of Spain, with a mean buoy position, computed from the archived Global Positioning System (GPS) records, of approximately 36.7349° N and 2.2081° W. Two non-contiguous observation windows were analyzed: 17 July–5 October 2025 and 16 May–23 July 2026. Figure 1 shows the deployment location.

The observing system consisted of a Spotter buoy equipped with a Smart Mooring system (Sofar Ocean Technologies, Inc., San Francisco, CA, USA). The Spotter measures three-dimensional surface displacement at a sampling rate of 2.5 Hz over a nominal wave-frequency range of 0.03–0.8 Hz. The embedded processing system provides Hs, peak wave period (Tp), mean wave period, peak and mean wave directions, and directional-spreading parameters [13,14]. Atmospheric pressure was measured using the onboard barometer, for which the manufacturer reports an accuracy of ±0.5 mbar (±0.5 hPa) at 25 °C and a measurement range of 700–1100 hPa [13].

Subsurface seawater temperature was measured using two Sofar Temperature Sensors integrated into the Smart Mooring and installed at 5 m (T5m; sensor position 1) and 10 m (T10m; sensor position 2). The Smart Mooring configuration allows temperature sensor nodes to be placed at different positions along the mooring cable. The sensors have a manufacturer-reported accuracy of ±0.1 °C, a resolution of 0.02 °C, a measurement range from −5 to 50 °C, and a maximum operating depth of 50 m [15].

Wind speed and direction were not measured using an onboard anemometer. Instead, the Spotter estimates wind from the equilibrium range of the high-frequency tail of the wave spectrum, with direction inferred from wave direction within that range [16]. These outputs were therefore treated as wave-spectrum-derived estimates rather than direct meteorological measurements. The distinction is relevant in coastal environments because the accuracy of the estimates may depend on fetch, wave development, directional alignment between wind and waves, and temporal variability in the wind field [17].

The measurement systems and variables considered in the analysis are summarized in Table 1.

### 2.2. External Products

External products were used as gridded consistency references rather than as ground-truth calibration standards. For each product, values were spatially interpolated to the mean buoy position using inverse-distance weighting of the nearest valid marine grid cells. All timestamps were expressed in Coordinated Universal Time (UTC), and buoy and external-product data were matched by UTC calendar day.

Seawater potential temperature was obtained from the Mediterranean Sea Physics Analysis and Forecast product (MEDSEA_ANALYSISFORECAST_PHY_006_013), using the daily dataset cmems_mod_med_phy-tem_anfc_4.2km_P1D-m. This Level-4 model product has a horizontal resolution of 0.042° and 141 vertical levels [18]. For all gridded external products, values were spatially interpolated to the mean buoy position using inverse-distance weighting of the nearest valid marine grid cells. The thetao variable was then extracted across the Copernicus Marine model levels bracketing the buoy sensors (3.17, 5.46, 7.92, and 10.54 m). Linear interpolation in the vertical was used to estimate model temperature at 5 and 10 m, allowing T5m and T10m to be compared with depth-matched Copernicus Marine fields. The remaining representativeness uncertainty is therefore related primarily to grid-cell support, unresolved coastal gradients, and vertical structure not captured at the buoy scale. The Copernicus product specifications confirm its 0.042° grid, 141 vertical levels, Level-4 processing, and availability of daily potential-temperature fields.

Wave parameters were obtained from the Mediterranean Sea Waves Analysis and Forecast product (MEDSEA_ANALYSISFORECAST_WAV_006_017), using the hourly dataset cmems_mod_med_wav_anfc_4.2km_PT1H-i. This Level-4 product provides hourly wave fields at a horizontal resolution of 0.042° [19]. The extracted variables were significant wave height (VHM0), peak wave period (VTPK), mean wave period (VTM02), and mean wave direction (VMDR). Scalar wave variables were aggregated using arithmetic daily means, whereas wave direction was aggregated using circular averaging. The official product documentation identifies it as the hourly 1/24° Mediterranean wave product and describes its WAve Model (WAM)-based forecasting system.

Wind data were obtained from the Global Ocean Hourly Sea Surface Wind and Stress from Scatterometer and Model product (WIND_GLO_PHY_L4_NRT_012_004), using the dataset cmems_obs-wind_glo_phy_nrt_l4_0.125deg_PT1H. This Level-4 product combines scatterometer observations and ECMWF operational model fields and provides hourly 10 m stress-equivalent wind components at a horizontal resolution of 0.125° [20]. Wind speed was calculated from the eastward and northward components, and wind direction was expressed using the meteorological “from” convention. Daily wind speed was obtained by arithmetic averaging of the hourly speed magnitudes, whereas daily direction was obtained using circular averaging. The product represents a gridded stress-equivalent wind field and is therefore not directly equivalent to the wave-spectrum-derived wind estimates provided by the Spotter.

Atmospheric pressure was obtained from the hourly ERA5 single-level dataset [9]. ERA5 provides hourly atmospheric reanalysis fields on a regular 0.25° × 0.25° latitude–longitude grid. Mean sea-level pressure was spatially interpolated to the mean buoy position from the available surrounding ERA5 grid points, converted from Pa to hPa, and aggregated using arithmetic daily means. The ERA5 series was used to assess temporal and synoptic consistency with the buoy barometer, rather than as a laboratory pressure calibration standard.

The external products and variables are summarized in Table 2.

### 2.3. Quality-Assessment and External-Consistency Workflow

Data processing, statistical analyses, and external-consistency metrics were performed using Python 3.12.6 (Python Software Foundation, Wilmington, DE, USA), with pandas 3.0.3, NumPy 2.4.5, and xarray 2026.4.0. Comparative figures were generated from the unified comma-separated value (CSV) outputs using Python 3.12.13, pandas 3.0.1, and Pillow 12.2.0. Copernicus Marine subsets were downloaded using the Copernicus Marine Toolbox 2.4.1 (Mercator Ocean International, Toulouse, France), whereas ERA5 mean sea-level pressure data were retrieved from the Copernicus Climate Data Store (CDS) using cdsapi 0.7.7 (European Centre for Medium-Range Weather Forecasts, Reading, UK).

The archived Spotter and Smart Mooring data streams were standardized to UTC and organized into channel-specific time series before aggregation. Duplicate Spotter timestamps were resolved by retaining the most complete record, whereas duplicate Smart Mooring timestamp–sensor-position records were averaged before reshaping the two temperature channels. Missing observations were retained as missing values, and no gap filling or temporal interpolation was applied. The same processing and quality-assessment procedure was applied to both observation windows.

Temporal gaps were identified as diagnostic interruptions when the interval between consecutive valid observations exceeded three times the expected sampling interval for the corresponding channel.

Completeness was computed independently for each channel as observed samples divided by expected samples, where expected samples equal the number of UTC days multiplied by the expected samples per day. The expected sampling intervals were 20 min for temperature and battery voltage (72 samples per day), 30 min for wave and wind variables (48 samples per day), and 60 min for atmospheric pressure (24 samples per day). Daily values were retained for external comparison only when at least 75% of the expected observations for the corresponding channel and UTC day were available. Sensitivity tests using 50%, 75%, and 90% daily-completeness thresholds were also calculated.

QC was applied at record level before daily aggregation using a QARTOD-informed flagging logic with missing, pass, suspect, and fail outcomes [21,22,23,24]. Gross-range failures were treated as hard failures and would be excluded from daily means. Suspect records from site–month range, spike, rate-of-change, flat-line, and gap tests were retained and reported because they indicate records requiring diagnostic interpretation rather than automatic rejection. Internal temperature consistency was assessed from paired T5m and T10m daily means, with |T5m-T10m| > 0.5 °C as the manual-review threshold. Table 3 summarizes the implemented QC tests and thresholds.

QARTOD procedures requiring independent neighboring observations, redundant co-located sensors, or post-deployment calibration records were not implemented as automatic rejection tests because these reference data were not available for the buoy; these aspects were therefore treated through external-product comparison, residual-trend analysis, and diagnostic interpretation rather than record-level exclusion.

**Figure 2:**
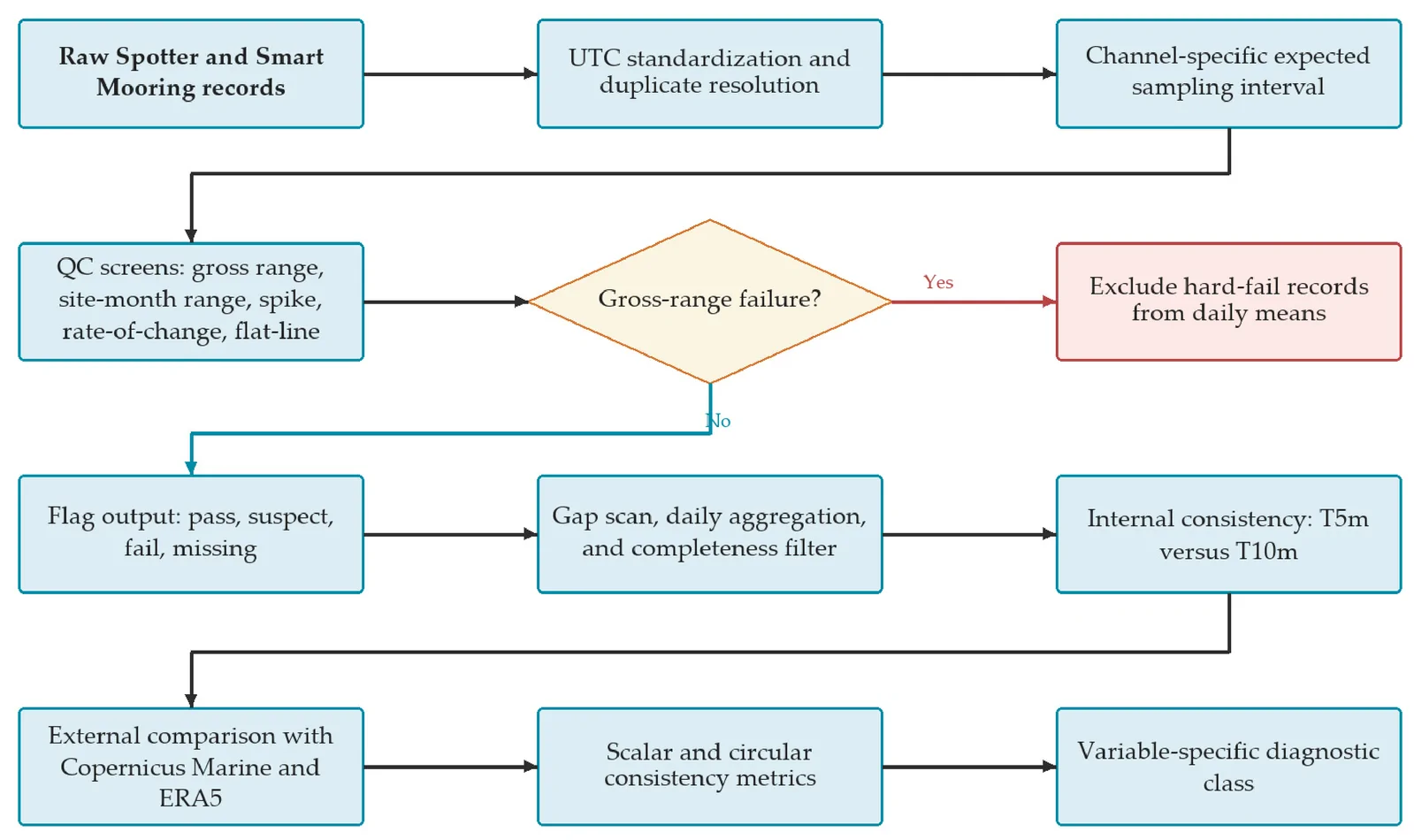
Workflow used for channel-specific quality assessment and external-consistency assessment of the C-GATA buoy observations.

Figure 2 summarizes the complete workflow, from archived data integration to channel-specific external-consistency assessment.

### 2.4. External-Consistency Metrics

For the primary scalar external-consistency variables, namely T5m, T10m, Hs, wave-spectrum-derived wind speed, and atmospheric pressure, the difference was defined as buoy minus external product. Mean bias, mean absolute error (MAE), root mean square error (RMSE), median difference, and Pearson’s correlation coefficient (r) were computed using only UTC days satisfying the channel-specific 75% completeness criterion. Monotonic trends in daily buoy-minus-product residuals were assessed using the Mann–Kendall test and Sen’s slope estimator for depth-matched T5m and T10m temperature comparisons and atmospheric pressure within each observation window. For directional variables, angular differences were wrapped to the interval from −180 deg to 180 deg before calculating circular mean bias, angular MAE, angular RMSE, and circular–circular correlation. For the wave-spectrum-derived wind channel, daily deep-water wave steepness was computed as S = 2*pi*Hs/(g*Tp^2) from buoy Hs and Tp, and days were grouped into window-specific steepness terciles to examine whether wind-speed differences depended on sea state. Peak wave period was retained as a diagnostic variable. It was not assigned a direct external-consistency class because the archived buoy field could not be unambiguously matched to the Copernicus Marine VTPK definition.

## 3. Results

### 3.1. Channel-Specific Completeness and Physical Plausibility

**Figure 3:**
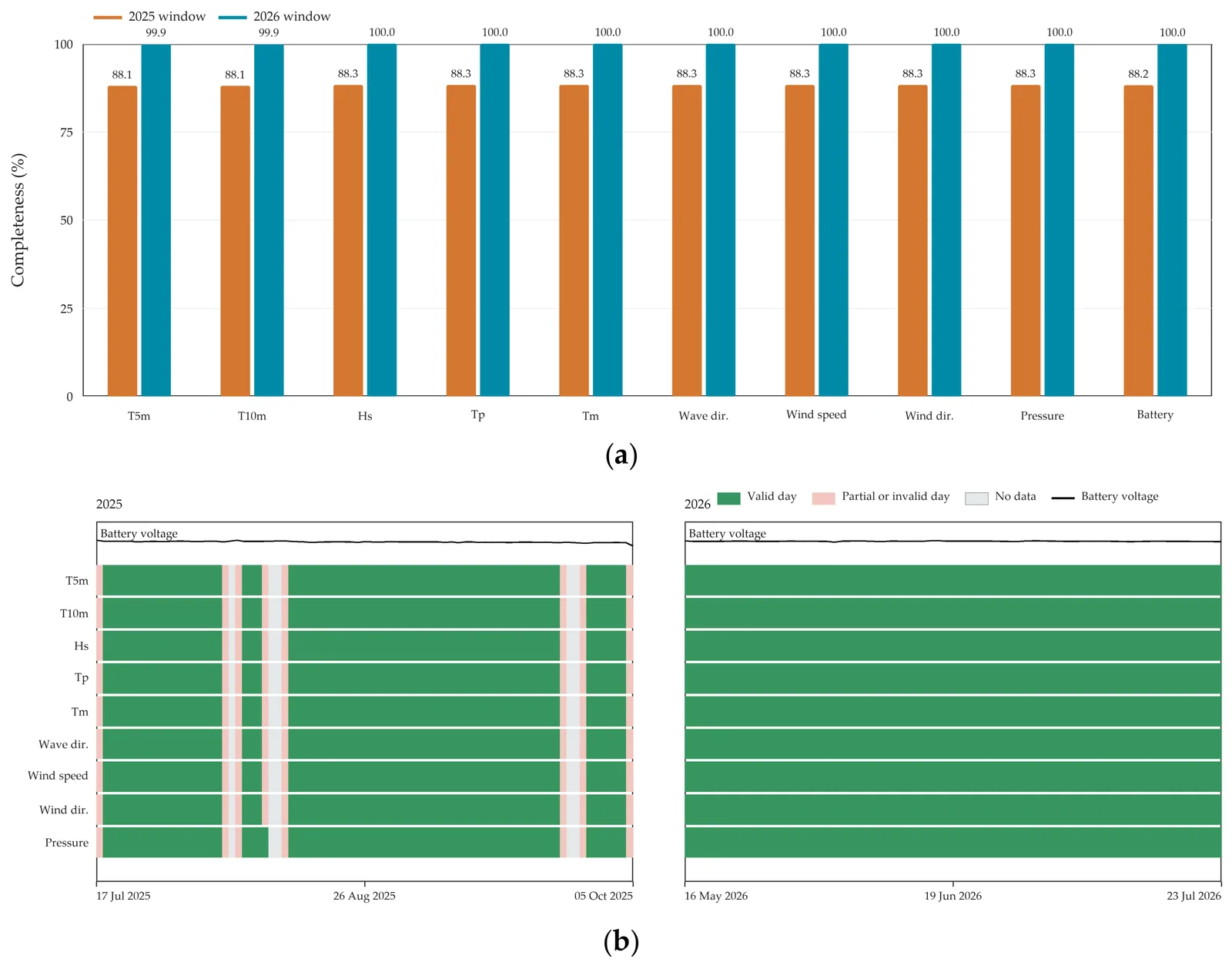
Channel-specific temporal availability during both observation windows: (**a**) completeness relative to the expected sampling interval, including battery voltage; (**b**) daily channel availability based on the 75% completeness criterion, with daily battery voltage overlaid.

Channel-specific completeness and daily availability for both observation windows are summarized in Figure 3 and Table 4. In 2025, the principal variables showed 88.1–88.3% completeness over the 81-day analysis window. The 75% daily-completeness criterion retained 68 valid days for T5m, T10m, waves, and wind, and 69 valid days for atmospheric pressure. In 2026, the common observation window comprised 69 UTC calendar days, with 99.88% completeness for T5m and T10m, 99.96% for battery voltage, and 100.00% for the wave, wind, and pressure channels. All 69 days satisfied the 75% criterion in the 2026 window.

Full channel-by-channel flag counts for both observation windows are provided in the Primary Data file qc_flag_summary_cgata_2025_2026.csv. The same file also reports observed_min and observed_max for each analyzed channel and observation window, documenting the margin between observed values and gross-range limits. No records failed the gross-range screen in either observation window. The observed ranges were within the sensor- or variable-specific physical limits for temperature, Hs, wind speed, pressure, and battery voltage. Suspect flags from the site–month range, spike, rate-of-change, and flat-line tests were retained for diagnosis rather than removed automatically. In 2025, data loss was concentrated in temporally coherent gap episodes on 5–7 August, 11–14 August, and 25–28 September, affecting both temperature channels and the wave, wind, pressure, and battery streams. In 2026, only two short temperature-gap episodes were identified; no gap above the three-interval threshold was found for waves, wind, pressure, or battery voltage.

### 3.2. Depth-Resolved Temperature Consistency

**Figure 4:**
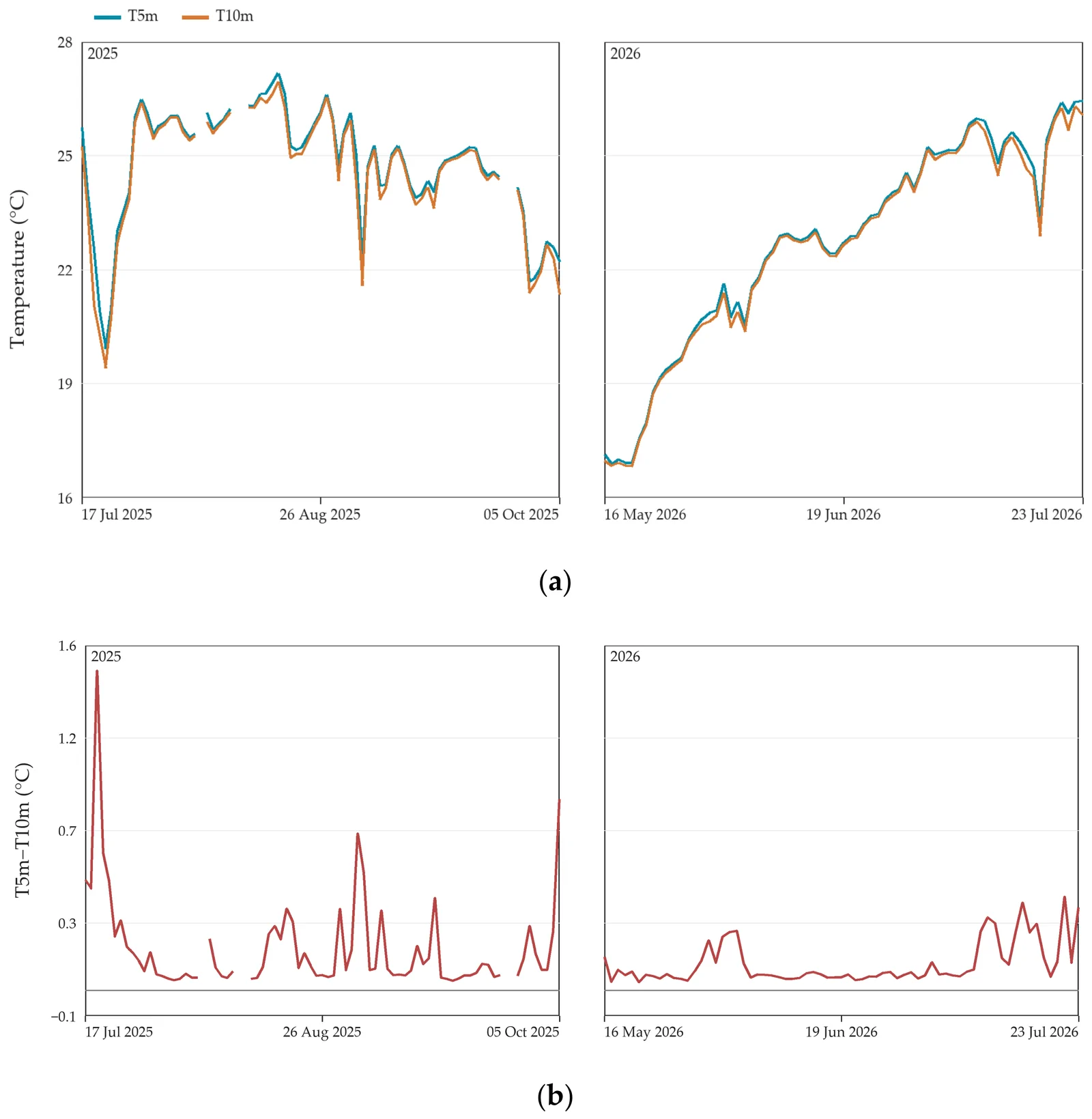
Depth-resolved temperature consistency of the C-GATA buoy during both observation windows: (**a**) daily seawater temperature recorded at 5 m and 10 m; (**b**) daily T5m-minus-T10m temperature difference.

The two temperature channels showed coherent daily variability in both observation windows, but with a persistent vertical offset (Figure 4). Deployment metadata identify sensor position 1 as the 5 m sensor (T5m) and position 2 as the 10 m sensor (T10m). A manual-review threshold of |T5m-T10m| > 0.5 °C flagged seven daily means in 2025 and none in 2026. The largest value occurred on 19 July 2025 (1.48 °C), during the early cooling episode, and was retained as a probable short-lived stratification or exposure event rather than treated as an automatic failure. In 2025, the mean T5m-minus-T10m difference was 0.20 °C and daily differences ranged from 0.05 to 1.48 °C. In 2026, the mean difference was 0.12 °C and daily differences ranged from 0.04 to 0.43 °C.

### 3.3. External-Consistency Assessment of Scalar Variables

Table 5 summarizes daily scalar external-consistency metrics. Temperature was compared against Copernicus Marine thetao linearly interpolated to the corresponding sensor depths. The results showed a strongly variable-dependent pattern. Atmospheric pressure and Hs provided the most robust external agreement across both observation windows, with low RMSE values and high correlations. T5m showed moderate agreement in 2025 and stronger agreement in 2026. In contrast, wind speed remained poorly comparable with Copernicus Marine Wind, despite applying the same temporal-validity filter.

### 3.4. Temperature at 5 m Versus Copernicus Marine

**Figure 5:**
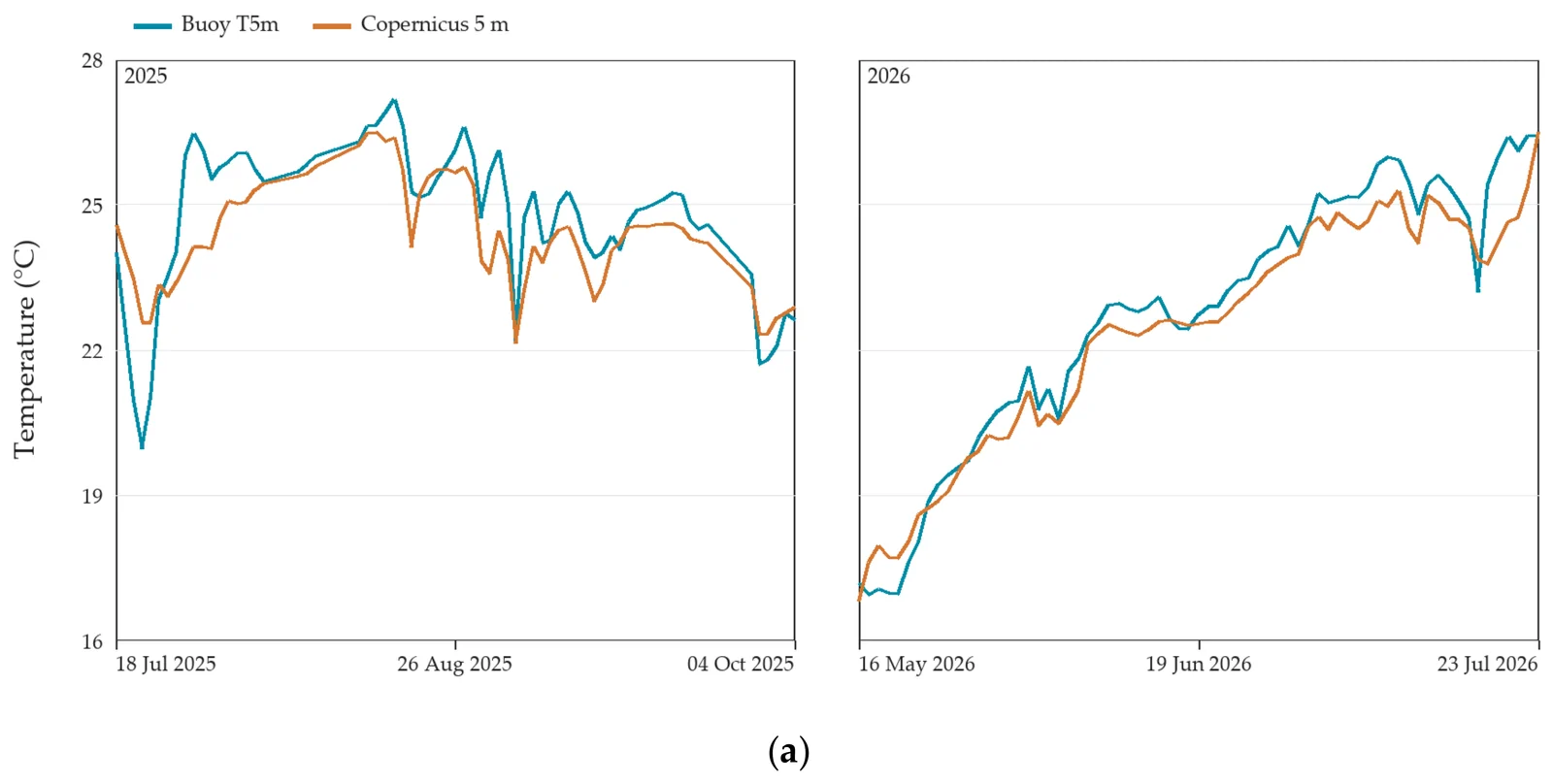

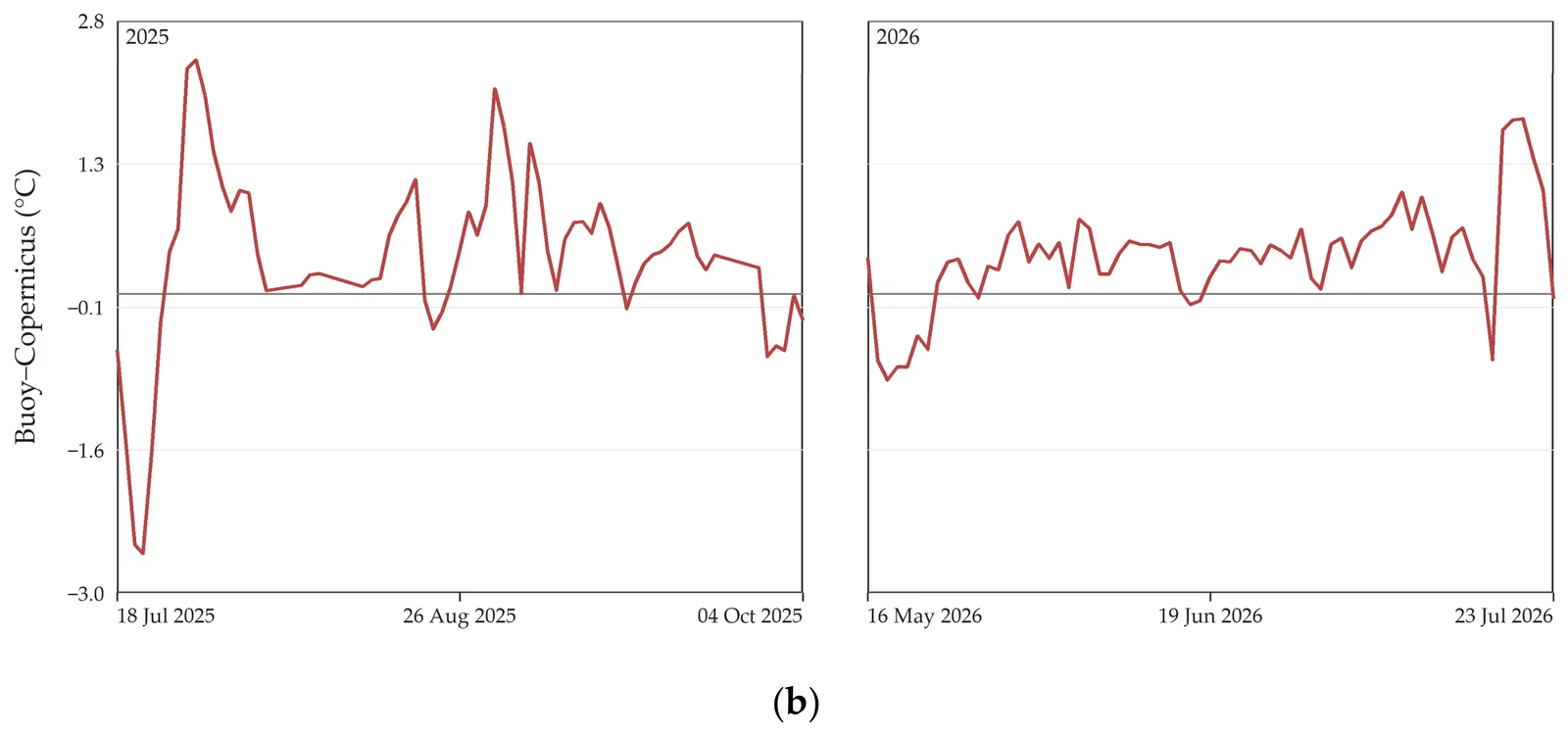
Daily temperature comparison between buoy T5m and Copernicus Marine thetao spatially interpolated to the buoy position and vertically interpolated to 5 m during both observation windows: (**a**) daily temperature series; (**b**) buoy T5m-minus-Copernicus Marine 5 m temperature difference.

The filtered daily comparison between buoy T5m and Copernicus Marine temperature interpolated to 5 m is shown in Figure 5. In 2025, the comparison yielded a mean bias of 0.38 °C, an MAE of 0.73 °C, an RMSE of 0.98 °C, and a correlation of 0.824. The largest negative difference occurred on 21 July 2025, during a rapid cooling that the buoy recorded more strongly than the gridded product, whereas the largest positive difference occurred on 27 July 2025. In 2026, agreement strengthened, with a buoy-minus-Copernicus bias of 0.37 °C, MAE of 0.52 °C, RMSE of 0.64 °C, and correlation of 0.987. T10m was also compared with Copernicus Marine temperature interpolated to 10 m (Table 5), yielding RMSE values of 1.17 °C in 2025 and 1.13 °C in 2026. Remaining discrepancies may reflect local coastal variability, unresolved vertical structure within the model layer, sensor exposure effects, or grid-cell representativeness.

### 3.5. Significant Wave Height and Wave Diagnostics

**Figure 6:**
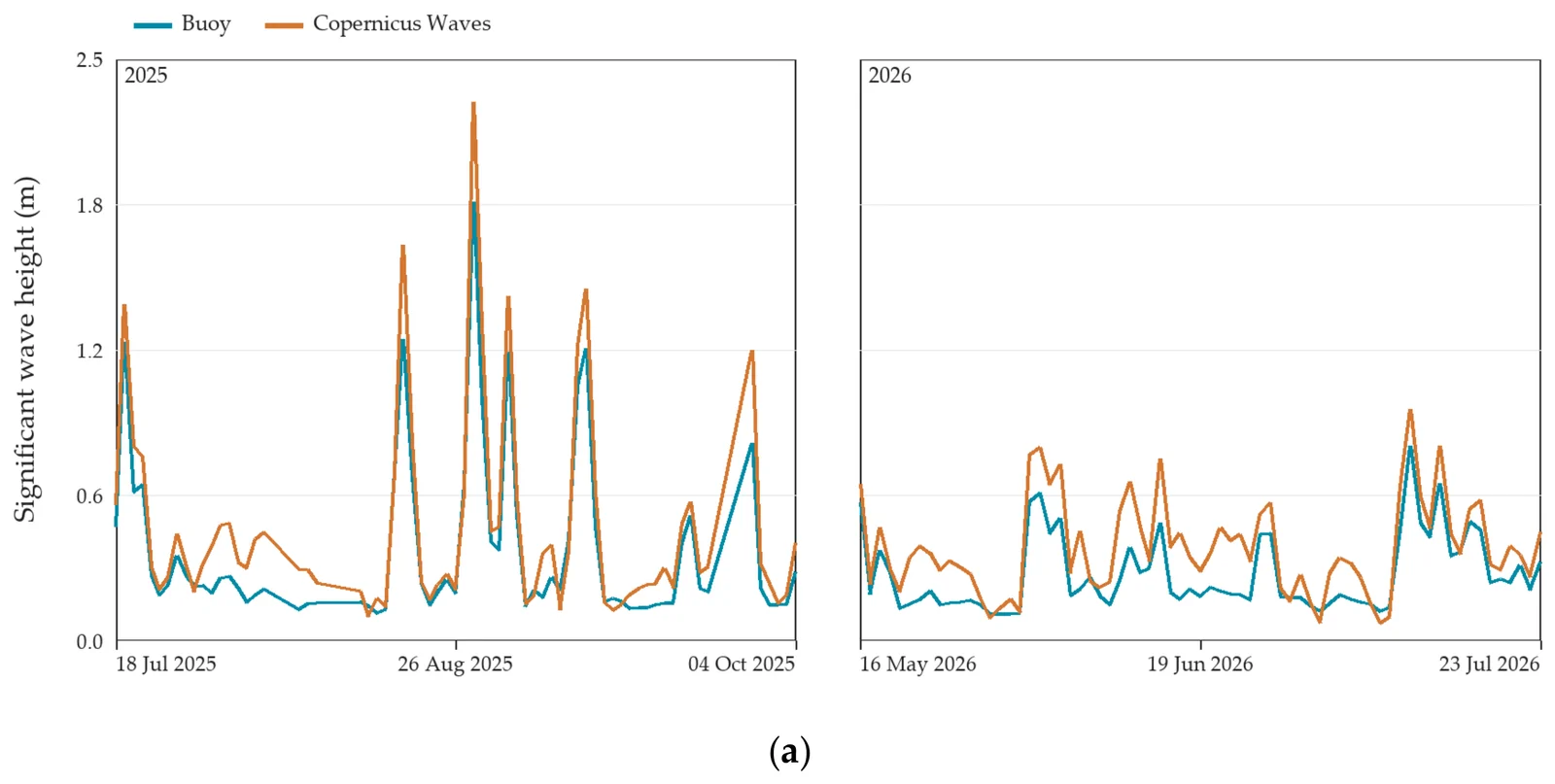

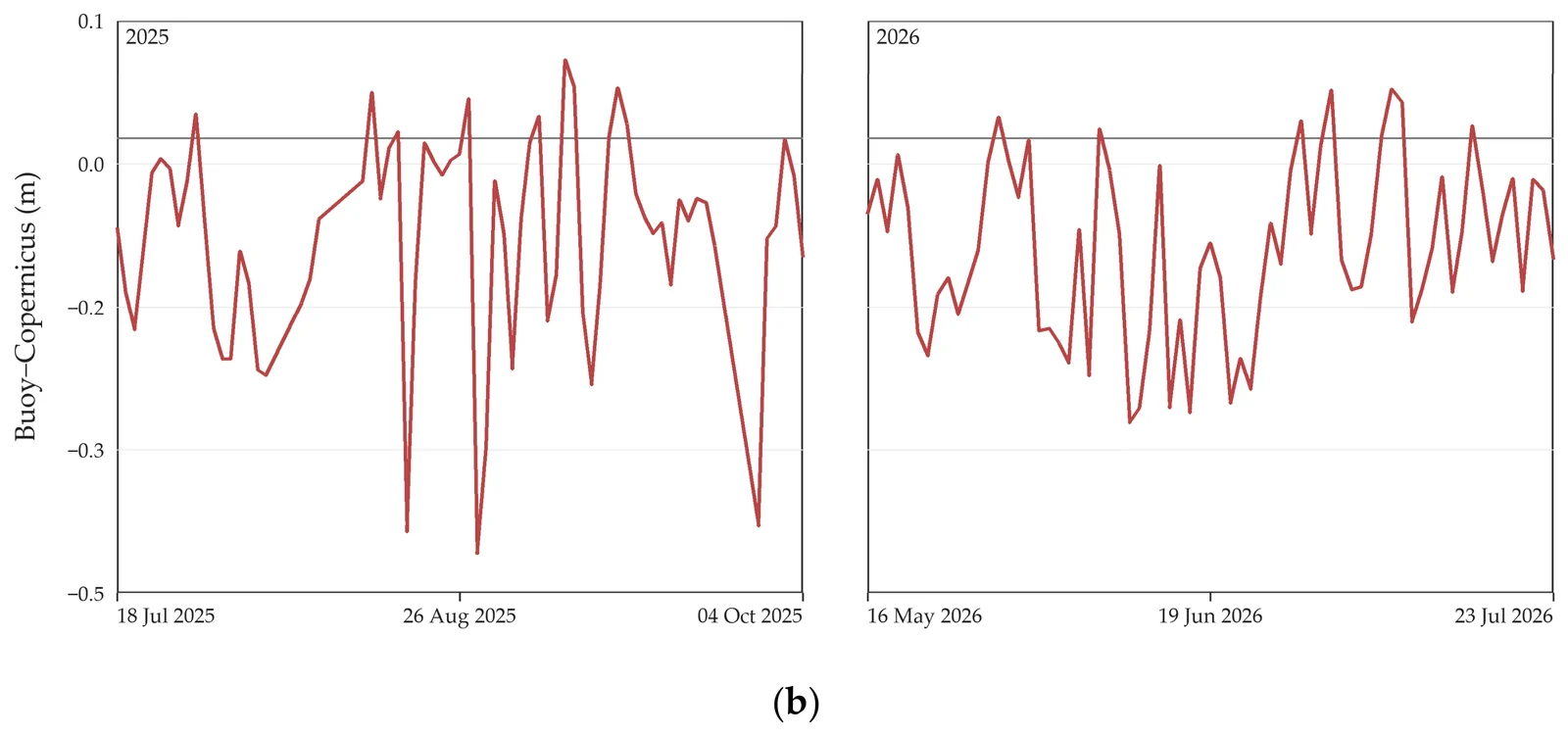
Daily significant wave height comparison between the C-GATA buoy and Copernicus Marine Waves VHM0 during both observation windows: (**a**) daily significant wave height series; (**b**) buoy-minus-Copernicus Marine Waves difference.

The daily comparison between buoy Hs and Copernicus Marine Waves VHM0 is shown in Figure 6. Significant wave height showed strong agreement after temporal filtering in both observation windows, with a consistent negative buoy-minus-Copernicus bias of −0.10 m in 2025 and −0.11 m in 2026. RMSE remained low, at 0.15 m and 0.14 m, respectively, and correlation was very high in 2025 (r = 0.981) and strong in 2026 (r = 0.904). These results identify Hs as the most robust wave variable for external-consistency assessment in this dataset. Peak wave period was not assigned a direct external-consistency class because the archived buoy field could not be unambiguously matched to the Copernicus Marine VTPK definition. Mean wave period and wave direction were therefore interpreted only as secondary diagnostics.

### 3.6. Wind-Speed Comparison

**Figure 7:**
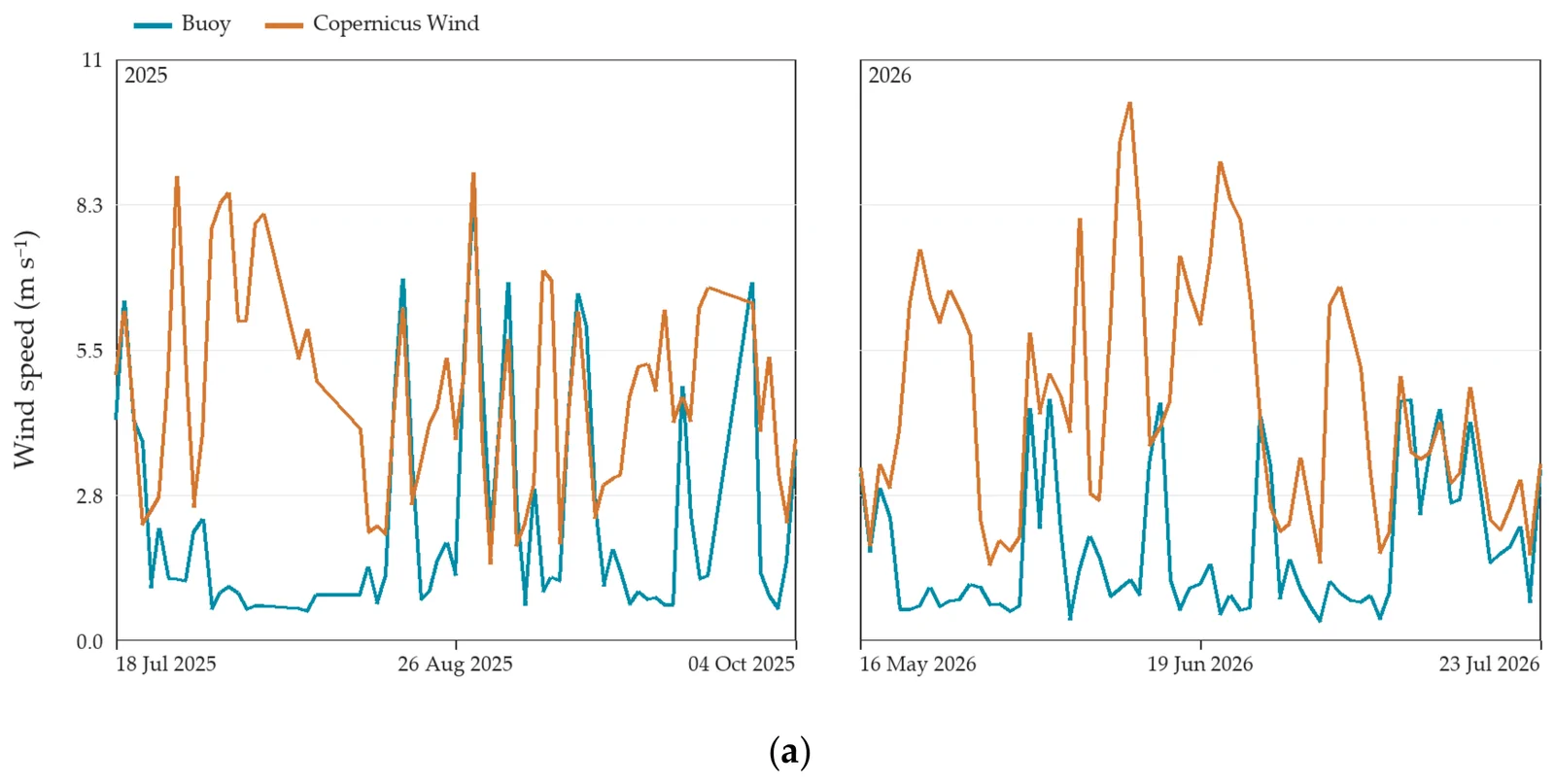

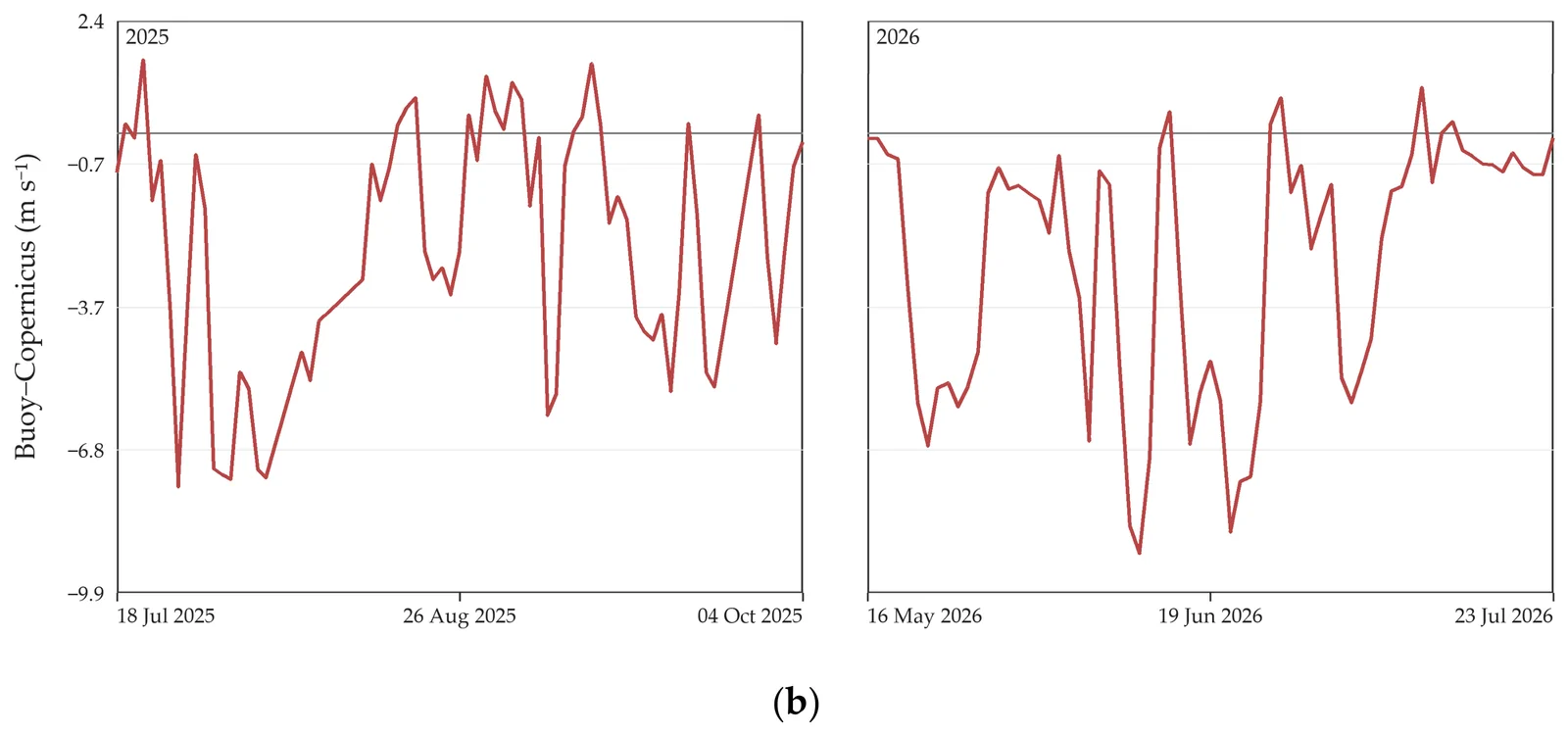
Daily wave-spectrum-derived wind-speed comparison between the C-GATA buoy and Copernicus Marine Wind during both observation windows: (**a**) daily wind-speed series; (**b**) buoy-minus-Copernicus Marine Wind difference.

The daily comparison between buoy wave-spectrum-derived wind speed and the IDW-interpolated Copernicus Marine stress-equivalent wind field is shown in Figure 7. Direct comparability remained limited in both observation windows. The mean buoy-minus-Copernicus bias was −2.36 m s^−1^ in 2025 and −2.77 m s^−1^ in 2026, with MAE values of 2.65 and 2.85 m s^−1^ and RMSE values of 3.51 and 3.88 m s^−1^, respectively. Correlation was low in 2025 (r = 0.108) and negative in 2026 (r = −0.166). The disagreement was strongly dependent on sea state: when daily wave steepness was grouped into terciles, mean absolute wind-speed differences decreased to 0.85 m s^−1^ in 2025 and 0.72 m s^−1^ in 2026 in the upper-steepness tercile, compared with 3.35–3.81 m s^−1^ and 3.44–4.39 m s^−1^ in the lower and middle terciles. The wind channel is therefore treated as a wave-state-dependent diagnostic rather than as a direct anemometer-equivalent measurement.

### 3.7. Atmospheric-Pressure Comparison

**Figure 8:**
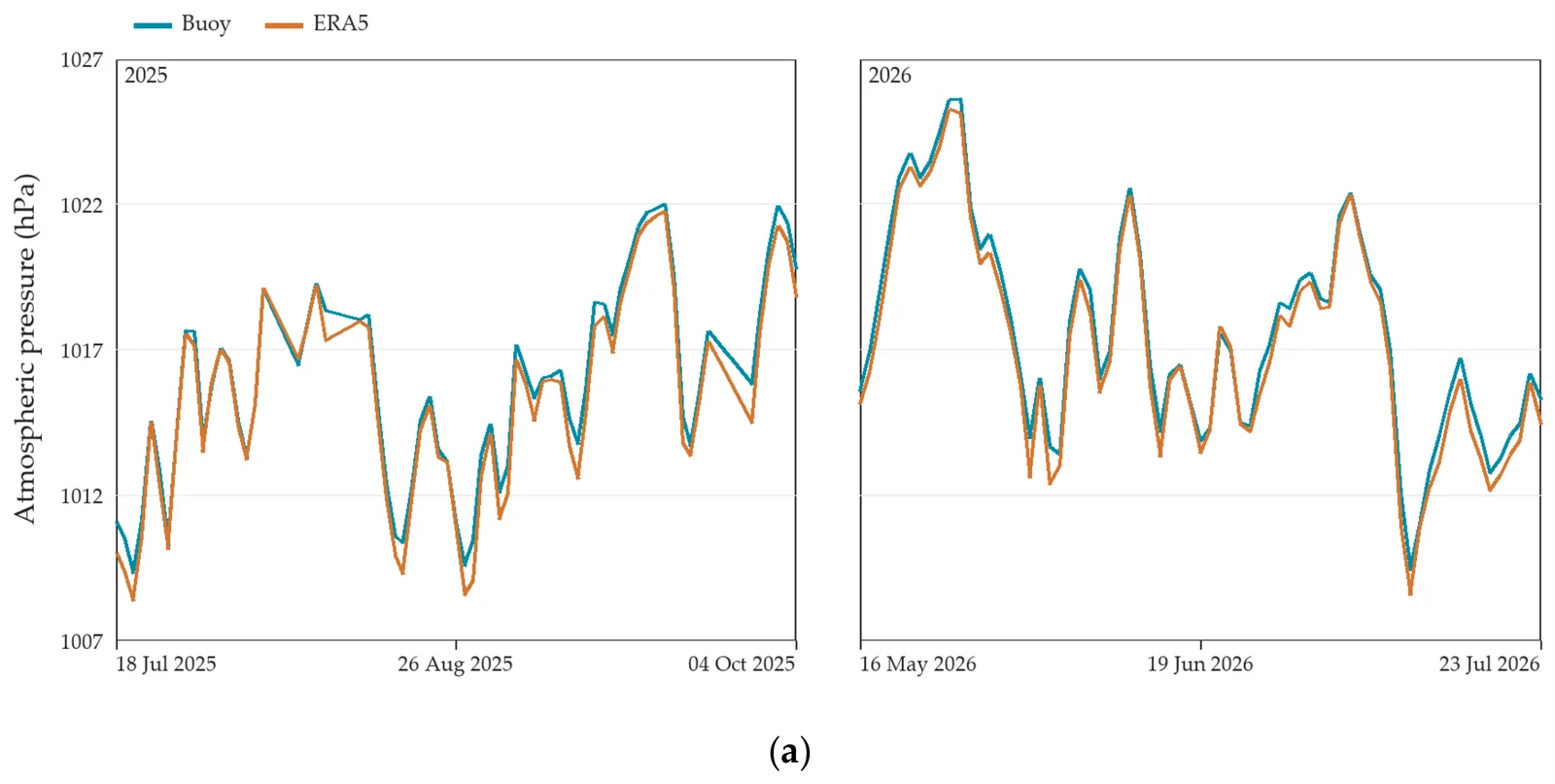

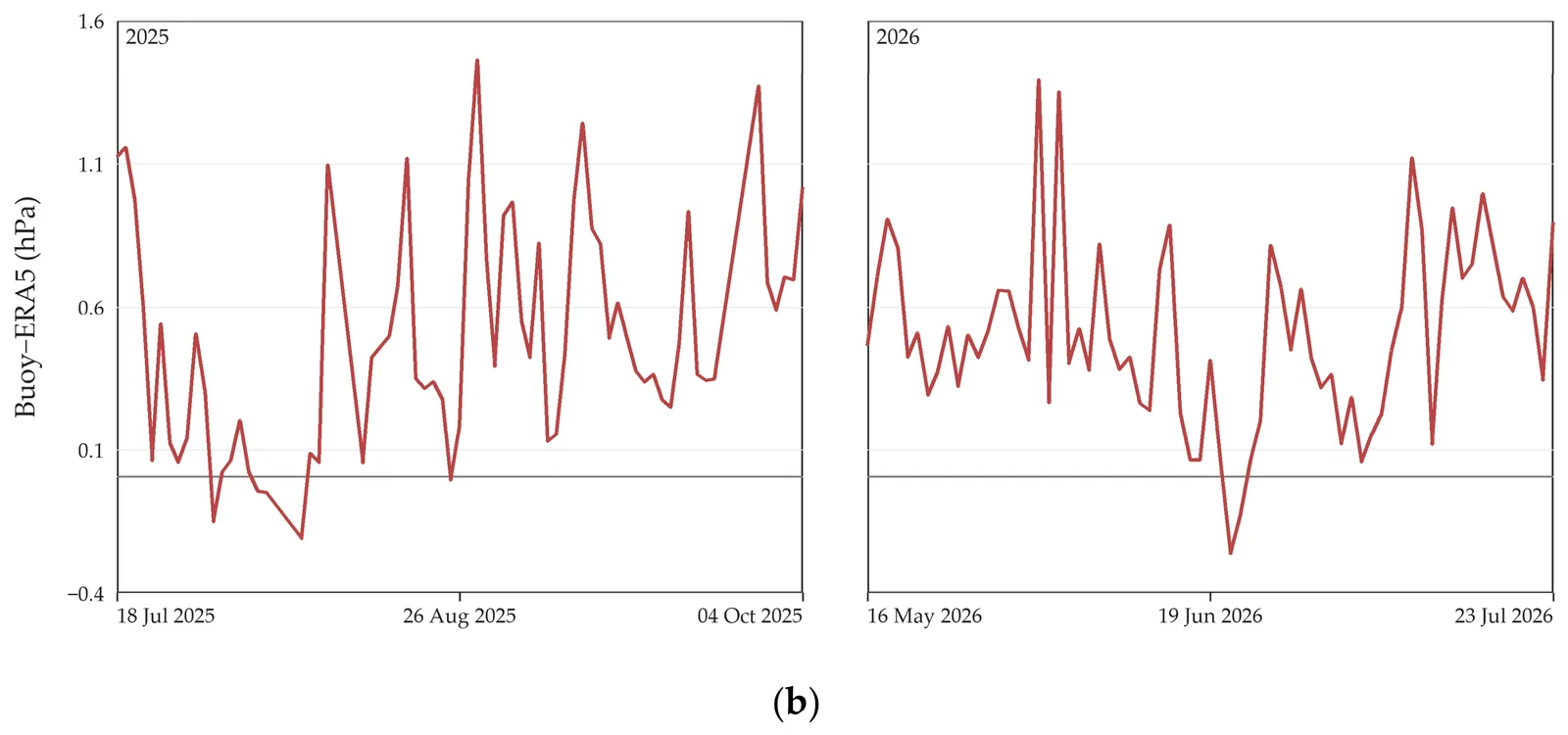
Daily atmospheric-pressure comparison between the C-GATA buoy and ERA5 mean sea-level pressure during both observation windows: (**a**) daily atmospheric-pressure series; (**b**) buoy-minus-ERA5 pressure difference.

The daily comparison between buoy atmospheric pressure and ERA5 mean sea-level pressure is shown in Figure 8. Because the barometer is located close to sea level, any hydrostatic height correction would be small relative to the observed synoptic variability. Atmospheric pressure showed very strong agreement with ERA5 in both observation windows. In 2025, the filtered comparison retained 69 valid days and produced a mean buoy-minus-ERA5 bias of 0.49 hPa, an MAE of 0.50 hPa, an RMSE of 0.63 hPa, and a correlation of 0.994. In 2026, the values were 69 valid days, a bias of 0.50 hPa, an MAE of 0.51 hPa, an RMSE of 0.59 hPa, and a correlation of 0.997.

**Figure 9:**
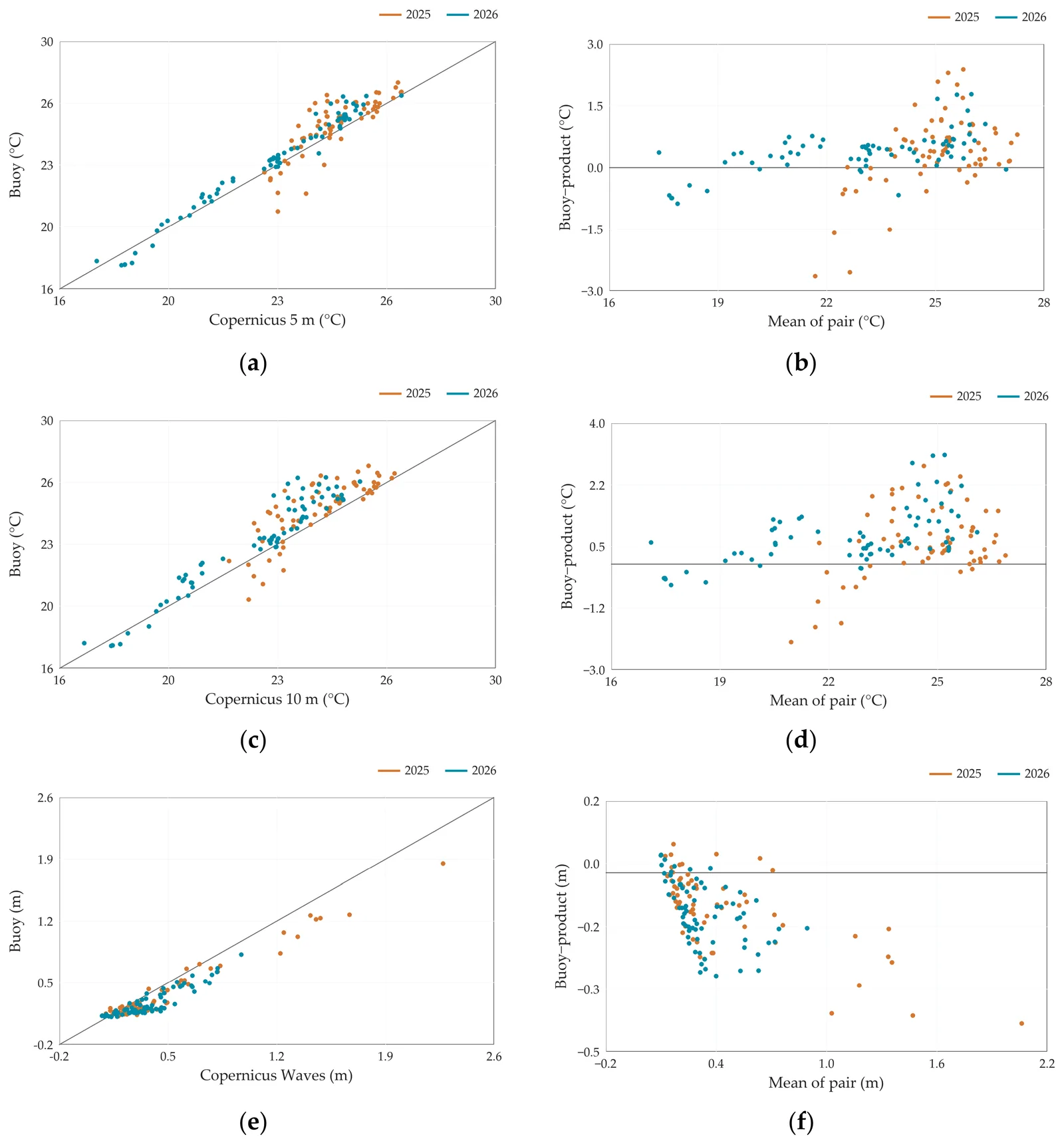

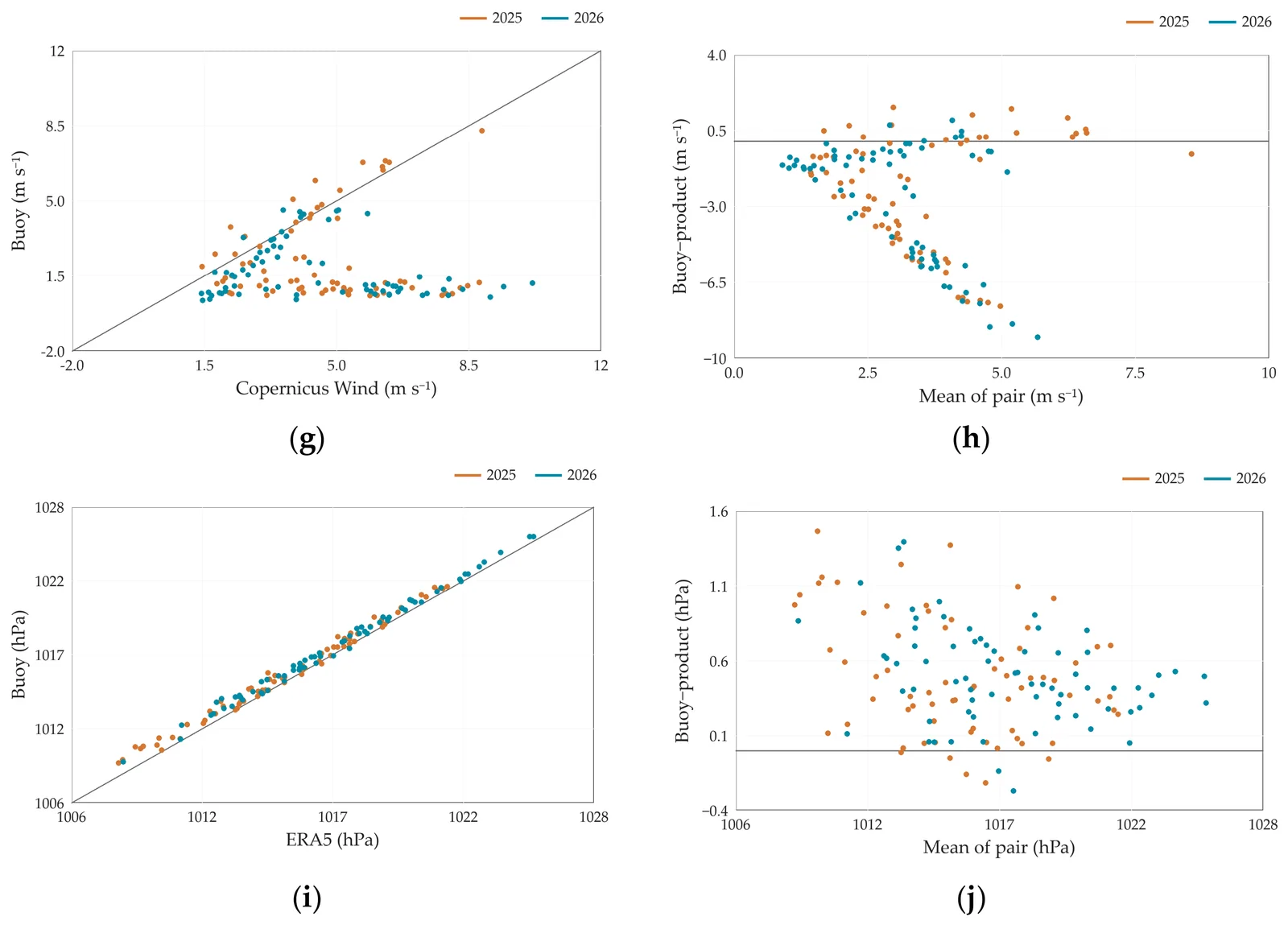
Scatter and agreement plots for scalar external-consistency variables after applying the 75% daily-completeness filter. Panels (**a**,**c**,**e**,**g**,**i**) show daily buoy values against external products with the 1:1 line for T5m, T10m, Hs, wave-spectrum-derived wind speed, and atmospheric pressure, respectively. Panels (**b**,**d**,**f**,**h**,**j**) show buoy-minus-product differences against the pairwise daily mean for the same variables. Orange points correspond to 2025 and blue points to 2026. Gridded products were spatially interpolated to the mean buoy position; temperature panels use Copernicus Marine thetao vertically interpolated to the corresponding buoy-sensor depth.

Scatter and agreement plots for the primary scalar comparisons are shown in Figure 9.

### 3.8. Directional Diagnostics

Directional comparisons are summarized in Table 6. Before interpreting the directional biases, alternative convention transformations were tested, including a 180 deg reversal of the buoy direction, a 180 deg reversal of the external-product direction, and a mirrored product direction around north. The documented convention produced the lowest angular MAE for both wave direction and wind direction in both windows, indicating that a simple from/to or clockwise/counter-clockwise convention correction did not remove the large biases. Mean wave direction showed circular biases of 45.03 deg in 2025 and 40.63 deg in 2026, with angular RMSE values of 65.96 deg and 60.39 deg, respectively. Wind direction showed larger circular biases of 74.06 deg in 2025 and 67.73 deg in 2026, with angular RMSE values of 93.03 deg and 88.90 deg, respectively.

### 3.9. Variable-Specific Diagnostic Classification

The channel-specific results were synthesized into a variable-specific diagnostic classification (Table 7). Because the variables have different units, physical meanings, and comparability with gridded products, no universal RMSE, bias, or correlation thresholds were applied; instead, Table 7 defines variable-specific criteria for strong agreement, moderate-to-good agreement, diagnostic-only use, and high-confidence interpretation. This classification combines temporal completeness, gross-range and suspect-flag outcomes, internal temperature consistency, external agreement metrics, and unresolved variable-definition or representativeness issues. A high-confidence classification required high temporal completeness, no gross-range failures, reproducible agreement in both windows, and strong external agreement using variable-specific thresholds. Diagnostic-only classifications were assigned to variables with high completeness but unresolved definition, directional, or representativeness limitations.

## 4. Discussion

The C-GATA buoy should not be treated as a single externally consistent or inconsistent platform; instead, each channel requires a specific diagnosis. This is consistent with ocean-observing best-practice recommendations, in which metadata, interoperability, traceability, and fit-for-purpose assessment are as important as numerical agreement with an external product [11,12,25].

The completeness analysis is also important. Percentages computed from the merged irregular master table may underestimate some channels because streams with different sampling intervals are combined on a row-by-row basis. Channel-specific expected sampling provides a more defensible measure of temporal completeness, consistent with ocean-observing data-management approaches that preserve sensor-level metadata, provenance, and QC information [26]. The contrast between the 2025 and 2026 windows also shows that the workflow can separate true temporal data loss from variable-specific agreement issues.

The 50%/75%/90% sensitivity analysis showed that the variable ranking was not an artifact of the selected 75% filter. In 2025, changing the threshold from 75% to 50% added only three days to the scalar comparisons, while changing to 90% left temperature, Hs, and wind unchanged and reduced pressure by one day. In 2026, all three thresholds retained the same 69 valid days for the principal channels.

The strongest and most reproducible results were obtained for atmospheric pressure and Hs. Pressure RMSE remained below 1 hPa, and correlation remained close to 1 in both windows, indicating that the buoy pressure channel is consistent with large-scale synoptic variability as represented by ERA5 [9]. Similarly, the low Hs bias in both windows supports using the buoy Hs channel for daily sea-state monitoring during the analyzed periods, consistent with previous Mediterranean wave-product and Spotter-buoy comparison studies [10,14,27]. These agreements should nevertheless be interpreted as external consistency checks, not as laboratory calibration.

The pressure residual analysis indicated a small, reproducible positive offset rather than a broad inconsistency. Mann–Kendall testing showed a weak increasing pressure residual in 2025 (Sen slope = 0.0065 hPa day^−1^, *p* = 0.015) but no residual trend in 2026 (Sen slope = 0.0003 hPa day^−1^, *p* = 0.930). The similar mean offsets in both windows (+0.49 and +0.50 hPa) are comparable to the manufacturer-reported barometer accuracy and are therefore a small residual offset.

The temperature comparison illustrates why disagreement with gridded products can be scientifically informative. Because T5m and T10m are physically distinct channels, the external comparison used Copernicus Marine temperature linearly interpolated to 5 and 10 m. The 2025 window contained an early cooling event recorded more strongly by the buoy than by Copernicus Marine, whereas the 2026 window showed stronger daily agreement during a seasonal warming period. These differences may reflect local coastal dynamics, unresolved vertical structure within the gridded model cell, sensor exposure, or grid-cell representativeness [28]. Interpretation of these temperature differences should therefore consider local wind forcing, upwelling, frontal dynamics, and bathymetry–current interactions in the Cabo de Gata–Alboran Sea sector as longer deployments become available [29,30].

The temperature residual trends do not by themselves prove biofouling. No temperature gross-range or flat-line failures were detected, and the strongest 2026 residual trend occurred during a seasonal warming period, when coastal stratification and grid-cell representativeness can also produce an increasing buoy-minus-model difference. The result therefore supports continued metadata and field-reference improvement rather than retrospective correction of the buoy record.

The wind-speed disagreement should be interpreted as a reproducible diagnostic outcome rather than as a simple instrument failure. Poor comparability persisted in the 2026 window despite near-complete temporal coverage, indicating that missing data were not the main cause. Because the buoy wind output is derived from the equilibrium range of the high-frequency directional wave spectrum [16,17], disagreement can arise when local seas are not in equilibrium with the instantaneous wind, when swell or coastal fetch limitations influence the spectrum, or when the gridded stress-equivalent wind field represents a larger-scale forcing [27]. A height correction alone would therefore not be sufficient; an independent anemometer or coastal-station comparison would be required before using the channel as a calibrated wind indicator.

The wave-steepness diagnostic supports this interpretation. In the upper wave-steepness tercile, mean absolute differences between the buoy wave-derived wind estimate and Copernicus Marine Wind decreased to 0.85 m s^−1^ in 2025 and 0.72 m s^−1^ in 2026. In the lower and middle terciles, the corresponding ranges were 3.35–3.81 m s^−1^ in 2025 and 3.44–4.39 m s^−1^ in 2026. This pattern indicates that the channel is more consistent with gridded wind under developed wave conditions and less representative when the local wave spectrum is not in equilibrium with the wind field.

The main limitations remain the short, non-contiguous observation windows and incomplete deployment-specific metadata. Adding a second observation window reduces dependence on a single window but does not eliminate the need for complete mounting, calibration, and sensor-exposure information. Future work should extend the time series, document complete mounting and calibration metadata, add independent in situ references where possible, and test sensitivity to high-frequency versus daily aggregation, consistent with current ocean-observing best-practice recommendations [11,12,25].

## 5. Conclusions

This study provides a channel-specific workflow for data quality assessment, representativeness analysis, and external-consistency assessment of the C-GATA coastal buoy near Cabo de Gata, applied to two observation windows in 2025 and 2026. The workflow documents available instrument metadata, recalculates completeness based on the expected sampling interval, applies QARTOD-informed gross-range, site–month range, spike, rate-of-change, flat-line, gap, and internal-consistency checks, filters daily external-consistency metrics by temporal completeness, and separates scalar comparison metrics from circular directional diagnostics.

The results support high confidence in daily atmospheric pressure and Hs, moderate-to-strong external consistency for depth-matched T5m, and diagnostic use only for wave-spectrum-derived wind unless independent wind observations are available. Peak wave period and directional variables should also be used diagnostically until variable definitions, directional offsets, and independent field references are resolved.

The main contribution is a transferable diagnostic framework for identifying which channels from a coastal buoy deployment are ready for environmental interpretation and which require additional metadata, calibration, or assessment of representativeness.

## Figures and Tables

**Table 1:** Measurement systems and analyzed variables of the C-GATA buoy.

Analyzed Variable	Measurement System or Derivation Method	Installation and Relevant Specifications
Wave parameters	Spotter-embedded motion-sensing and wave-processing system	Integrated into the surface buoy. Three-dimensional displacement sampled at 2.5 Hz over a nominal frequency range of 0.03–0.8 Hz.
Seawater temperature	Two Sofar Temperature Sensors	Installed at 5 m (T5m; sensor position 1) and 10 m (T10m; sensor position 2). Accuracy: ±0.1 °C; resolution: 0.02 °C; range: −5 to 50 °C; maximum operating depth: 50 m.
Atmospheric pressure	Spotter onboard barometer	Integrated into the surface buoy. Accuracy: ±0.5 mbar at 25 °C; range: 700–1100 mbar.
Wind speed and direction estimates	Estimates derived from the high-frequency directional wave spectrum	Indirect estimates derived from wave measurements rather than direct measurements obtained using an anemometer.

**Table 2:** External products and variables used for external-consistency assessment.

External Source	Variables Used	Native Temporal/Spatial Resolution and Treatment
Copernicus Marine Mediterranean Sea Physics Analysis and Forecast	Sea water potential temperature (thetao)	Daily; 0.042 deg; 141 vertical levels. Temperature spatially interpolated to the mean buoy position by inverse-distance weighting of nearest valid marine grid cells, then vertically interpolated at 3.17, 5.46, 7.92, and 10.54 m to 5 and 10 m.
Copernicus Marine Mediterranean Sea Waves Analysis and Forecast	VHM0, VTPK, VTM02, and VMDR	Hourly; 0.042 deg. Scalar variables spatially interpolated to the mean buoy position by inverse-distance weighting; direction spatially interpolated using sine–cosine components; daily means/circular means.
Copernicus Marine Global Ocean Hourly Sea Surface Wind and Stress	Eastward and northward stress-equivalent wind components	Hourly; 0.125 deg; 10 m stress-equivalent wind components spatially interpolated to the mean buoy position by inverse-distance weighting. Speed and meteorological direction derived from IDW-interpolated components and aggregated daily.
ERA5 hourly data on single levels	Mean sea-level pressure	Hourly; 0.25 deg; mean sea-level pressure spatially interpolated to the mean buoy position from available surrounding grid points, converted from Pa to hPa and averaged daily.

**Table 3:** QARTOD-informed QC tests implemented for the C-GATA buoy revision. Thresholds generate diagnostic flags unless explicitly identified as hard failures.

Implemented Step	QARTOD Correspondence	Threshold or Action in This Study
Duplicate resolution and gap scan	Timing/gap and syntax checks	Most complete duplicate retained; diagnostic gap when interval > 3× expected sampling interval.
Gross range	Required gross-range test	Hard fail outside sensor or physically admissible limits: −5–50 °C, 0–8 m Hs, 0–40 m s^−1^ wind speed, 700–1100 hPa pressure, 3.7–4.3 V battery.
Site–month expected range	Climatology/location-range screen	Suspect flag outside monthly nearest-product range expanded by variable-specific margins; retained for daily means.
Spike test	Strongly recommended spike test	Suspect flag when a single-step change exceeded the channel threshold and the time step was <= 2.1× the expected interval.
Rate-of-change test	Strongly recommended rate-of-change test	Suspect flag when hourly rate exceeded the channel threshold under regular sampling.
Flat-line test	Strongly recommended flat-line/attenuated-signal test	Suspect flag for repeated identical values over the minimum channel-specific sequence length.
T5m–T10m internal consistency	Multivariate/internal consistency diagnostic	Manual-review flag when absolute daily T5m-T10m difference exceeded 0.5 °C; flagged days were retained.

**Table 4:** Expected sampling interval, observed samples, and daily-validity sensitivity for the analyzed buoy channels. Expected samples equal analysis days multiplied by expected samples per day; valid-day columns report 50%/75%/90% daily-completeness thresholds.

Channel	Interval (min)	Expected/Day	2025 Observed/ Expected	2025 Valid Days 50/75/90	2026 Observed/ Expected	2026 Valid Days 50/75/90
T5m	20	72	5136/5832 (88.1%)	71/68/68	4962/4968 (99.9%)	69/69/69
T10m	20	72	5136/5832 (88.1%)	71/68/68	4962/4968 (99.9%)	69/69/69
Hs	30	48	3434/3888 (88.3%)	71/68/68	3312/3312 (100.0%)	69/69/69
Tp	30	48	3434/3888 (88.3%)	71/68/68	3312/3312 (100.0%)	69/69/69
Tm	30	48	3434/3888 (88.3%)	71/68/68	3312/3312 (100.0%)	69/69/69
Wave direction	30	48	3434/3888 (88.3%)	71/68/68	3312/3312 (100.0%)	69/69/69
Wind speed	30	48	3434/3888 (88.3%)	71/68/68	3312/3312 (100.0%)	69/69/69
Wind direction	30	48	3434/3888 (88.3%)	71/68/68	3312/3312 (100.0%)	69/69/69
Pressure	60	24	1717/1944 (88.3%)	71/69/68	1656/1656 (100.0%)	69/69/69
Battery	20	72	5146/5832 (88.2%)	71/68/68	4966/4968 (100.0%)	69/69/69

**Table 5:** Daily scalar external-consistency metrics for the 2025 and 2026 observation windows after applying the 75% channel-completeness criterion. Differences are computed as buoy minus external product; gridded products were spatially interpolated to the mean buoy position, and Copernicus Marine temperature was vertically interpolated to the corresponding buoy-sensor depth.

Window and Variable	External Product	n	Bias	MAE	RMSE	Median Diff.	Correlation
2025: Temperature at 5 m (°C)	Copernicus Marine Physics	68	0.38	0.73	0.98	0.41	0.824
2025: Temperature at 10 m (°C)	Copernicus Marine Physics	68	0.64	0.91	1.17	0.57	0.808
2025: Significant wave height, Hs (m)	Copernicus Marine Waves	68	−0.10	0.11	0.15	−0.09	0.981
2025: Wind speed (m s^−1^)	Copernicus Marine Wind	68	−2.36	2.65	3.51	−1.81	0.108
2025: Atmospheric pressure (hPa)	ERA5	69	0.49	0.50	0.63	0.42	0.994
2026: Temperature at 5 m (°C)	Copernicus Marine Physics	69	0.37	0.52	0.64	0.36	0.987
2026: Temperature at 10 m (°C)	Copernicus Marine Physics	69	0.79	0.87	1.13	0.61	0.964
2026: Significant wave height, Hs (m)	Copernicus Marine Waves	69	−0.11	0.12	0.14	−0.11	0.904
2026: Wind speed (m s^−1^)	Copernicus Marine Wind	69	−2.77	2.85	3.88	−1.29	−0.166
2026: Atmospheric pressure (hPa)	ERA5	69	0.50	0.51	0.59	0.46	0.997

**Table 6:** Circular directional diagnostics for the 2025 and 2026 observation windows. Angular differences are wrapped to the interval from −180 deg to 180 deg.

Window and Variable	External Product	n	Circular Bias (deg)	Angular MAE (deg)	Angular RMSE (deg)	Circular Correlation
2025: Mean wave direction	Copernicus Marine Waves	68	45.03	50.72	65.96	0.831
2025: Wind direction	Copernicus Marine Wind	68	74.06	77.47	93.03	0.703
2026: Mean wave direction	Copernicus Marine Waves	69	40.63	44.96	60.39	0.930
2026: Wind direction	Copernicus Marine Wind	69	67.73	70.65	88.90	0.810

**Table 7:** Variable-specific diagnostic classification for the C-GATA observation windows, using explicit completeness, QC, and external-consistency criteria.

Channel	Completeness/QC Result	External-Agreement Criterion	Observed Agreement	Diagnostic Class
Temperatures at 5 and 10 m	High completeness; no gross-range failures; seven T5m-T10m daily review flags in 2025 and none in 2026.	Moderate to good when r >= 0.75 and RMSE is <= 1.2 °C after depth matching.	T5m RMSE 0.98/0.64 °C; T10m RMSE 1.17/1.13 °C.	Usable with depth and representativeness caution.
Significant wave height	High completeness; no gross-range, spike, or rate-of-change failures.	Strong when |bias| <= 0.15 m, RMSE <= 0.20 m, and r >= 0.90 in both windows.	Bias −0.10/−0.11 m; RMSE 0.15/0.14 m; r 0.981/0.904.	High confidence for daily Hs.
Peak wave period	High completeness but frequent suspect flags in site-range, spike, rate-of-change, and flat-line tests.	Primary external-consistency classification not assigned because the archived buoy variable and VTPK definition could not be verified as equivalent.	Used in the wave-steepness diagnostic.	Definition review before direct comparison.
Mean wave direction	High completeness; no gross-range failures.	Diagnostic when circular correlation is moderate to high but angular bias/RMSE remains large.	Bias 45.03/40.63 deg; angular RMSE 65.96/60.39 deg.	Diagnostic only until directional offset is resolved.
Wind speed	High completeness but frequent flat-line flags in low wave-derived wind estimates.	Direct agreement with gridded wind is not expected unless sea-state dependence and an independent wind reference are considered.	Bias −2.36/−2.77 m s^−1^; RMSE 3.51/3.88 m s^−1^; r 0.108/−0.166. Upper wave-steepness terciles had mean absolute differences of 0.85/0.72 m s^−1^.	Wave-state-dependent diagnostic; independent wind reference required.
Wind direction	High completeness; no gross-range failures.	Diagnostic when angular error remains large after convention checks.	Bias 74.06/67.73 deg; angular RMSE 93.03/88.90 deg.	Diagnostic only.
Atmospheric pressure	High completeness; no gross-range, spike, or rate-of-change failures.	Strong when RMSE <= 1 hPa and r >= 0.99 in both windows.	Bias 0.49/0.50 hPa; RMSE 0.63/0.59 hPa; r 0.994/0.997.	High confidence for daily synoptic variability, with a small offset noted.

## Data Availability

The raw buoy files used in this study and the channel-level quality-control summary are provided as Primary Data.

## Abbreviations

The following abbreviations are used in this manuscript:

CDS	Copernicus Climate Data Store
C-GATA	Calibration and Geospatial Analysis of Temporal Oceanographic Anomalies
CSV	Comma-separated values
ECMWF	European Centre for Medium-Range Weather Forecasts
ERA5	Fifth-generation ECMWF atmospheric reanalysis
GPS	Global Positioning System
Hs	Significant wave height
MAE	Mean absolute error
QC	Quality control
QARTOD	Quality Assurance/Quality Control of Real-Time Oceanographic Data
RMSE	Root mean square error
T5m	Seawater temperature measured at 5 m depth
T10m	Seawater temperature measured at 10 m depth
Tp	Peak wave period
UTC	Coordinated Universal Time
VHM0	Copernicus Marine Waves significant wave height variable
VMDR	Copernicus Marine Waves mean wave direction variable
VTM02	Copernicus Marine Waves mean wave period variable
VTPK	Copernicus Marine Waves peak wave period variable
WAM	WAve Model
